# Volumetric Semantic Instance Segmentation of the Plasma Membrane of HeLa Cells

**DOI:** 10.3390/jimaging7060093

**Published:** 2021-06-01

**Authors:** Cefa Karabağ, Martin L. Jones, Constantino Carlos Reyes-Aldasoro

**Affiliations:** 1 giCentre, Department of Computer Science, School of Mathematics, Computer Science and Engineering, City, University of London, London EC1V 0HB, UK; cefa.karabag@city.ac.uk; 2 Electron Microscopy Science Technology Platform, The Francis Crick Institute, London NW1 1AT, UK; martin.jones@crick.ac.uk

**Keywords:** semantic instance segmentation, HeLa cells, plasma membrane

## Abstract

In this work, an unsupervised volumetric semantic instance segmentation of the plasma membrane of HeLa cells as observed with serial block face scanning electron microscopy is described. The resin background of the images was segmented at different slices of a 3D stack of 518 slices with 8192 × 8192 pixels each. The background was used to create a distance map, which helped identify and rank the cells by their size at each slice. The centroids of the cells detected at different slices were linked to identify them as a single cell that spanned a number of slices. A subset of these cells, i.e., the largest ones and those not close to the edges were selected for further processing. The selected cells were then automatically cropped to smaller regions of interest of 2000 × 2000 × 300 voxels that were treated as cell instances. Then, for each of these volumes, the nucleus was segmented, and the cell was separated from any neighbouring cells through a series of traditional image processing steps that followed the plasma membrane. The segmentation process was repeated for all the regions of interest previously selected. For one cell for which the ground truth was available, the algorithm provided excellent results in Accuracy (AC) and the Jaccard similarity Index (JI): nucleus: JI =0.9665, AC =0.9975, cell including nucleus JI =0.8711, AC =0.9655, cell excluding nucleus JI =0.8094, AC =0.9629. A limitation of the algorithm for the plasma membrane segmentation was the presence of background. In samples with tightly packed cells, this may not be available. When tested for these conditions, the segmentation of the nuclear envelope was still possible. All the code and data were released openly through GitHub, Zenodo and EMPIAR.

## 1. Introduction

In 1951, cervical cells extracted from a patient called Henrietta Lacks at Johns Hopkins Hospital were to become the first continuous cancer cell line [1]. The cells are widely known as *HeLa* cells (from Henrietta Lacks) and have become a centrepiece of biomedical research, spanning from AIDS [2] to toxicity [3] to Zika [4] and, of course, cancer. As of 2021, PubMed contained more than 110,000 entries related to HeLa cells (https://pubmed.ncbi.nlm.nih.gov/?term=HeLa+[all+fields]) (Accessed on 28 May 2021). Since the cells were removed and kept without the patient’s knowledge or consent, which was not required at that time, many ethical and legal issues have also followed the HeLa cells [5,6,7,8].

The observation of the cells and their characteristics such as shape, colours or size and their relationship to health or disease is probably as old as the studies by van Leeuwenhoek and Hooke [9]. Whist the cell structure and its organelles have been well known for many years, discoveries related to cell structure continue to appear in the scientific literature; searches in Google Scholar for terms such as cell structure and function (https://scholar.google.co.uk/scholar?as_ylo=2021&q=cell+structure+and+function (Accessed on 28 May 2021)) or cell structure and transport (https://scholar.google.co.uk/scholar?as_ylo=2021&q=cell+structure+and+transport (Accessed on 28 May 2021)) returned more than 50,000 entries in the first four months of 2021 alone.

**Figure 1:**
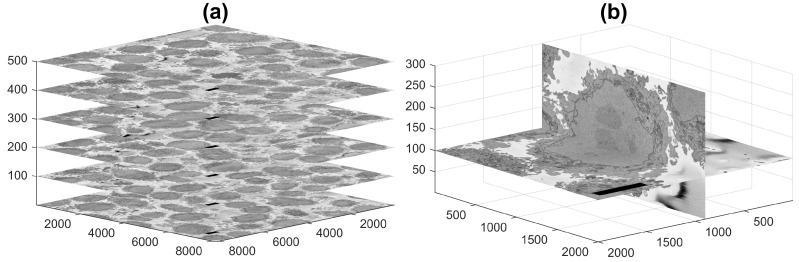
Representative slices of a 3D image stack acquired by Serial Block-face Scanning Electron Microscopy (SBF SEM) containing numerous HeLa cells. (**a**) Six of the stack of 518 of electron microscopy images. (**b**) For visualisation purposes, two slices of a HeLa cell image are presented orthogonal to each other. In both cases, the units of the axes are in voxels, and 5 μm scale bars are shown towards the left of all horizontal images.

Today, sophisticated instruments, such as Electron Microscopes (EMs), allow observation with significant resolution, far greater than those of conventional light and fluorescence microscopes, and in turn allow the observation of smaller structures and provide great detail of the larger ones. Additionally, three-dimensional observation is possible by cutting very thin sections of a fixed sample with an ultra-microtome diamond knife [10] and acquiring images of the top face as each section is removed. The slice acquisition process is known as *Serial Block-face Scanning EM* (SBF SEM) [11], and the output is illustrated in Figure 1a, where selected images are positioned in three dimensions according to their location in the volumetric stack. Figure 1b presents a zoom into a single cell. One image is presented horizontally, and one orthogonal slice, or *orthoslice*, which was obtained from 300 images, is displayed vertically.

Cells are normally kept in shape by the plasma membrane, a phospholipid bilayer membrane that separates the internal aqueous environment of the cell and its organelles from the external environment [12]. In addition, the nucleus and chromosomes are surrounded by another bilayer membrane, the nuclear envelope [13]. The study of cellular membranes has a long history, and the study and discovery of cell structures “keep biologists glued to their microscopes” [14]. Cellular membrane receptors are important in conditions such as Alzheimer’s disease [15], cancer [16] and Helicobacter pylori infection [17]. The geometry of the membranes is also important, for instance the shape [18,19], curvature [20,21] and protuberances [22] have been studied. The importance of the nuclear envelope in particular is related in processes such as viral infections [23], cancer [24] and cardiovascular function [25] and has been an area of research for a long time [26,27]. Therefore, algorithms that provide accurate segmentation of the membranes of a cell are of great importance as the visualisation and analysis of the membranes and shape of cells could provide clues to understand the health or disease of cells and their organs [28,29,30,31,32,33]. The reader is referred to Lombard [34] for a historical review of the cell membranes.

Segmentation can be understood as the process of partitioning images or volumes into homogeneous non-overlapping regions [35]; in the simplest case, one region is the background, and the other region is an object or the foreground. Semantic segmentation identifies the pixels as a series of classes or labels that have a particular meaning, such as a person, a car, a cell or an organ [36]. Going further, instance segmentation is the process of detecting and segmenting each distinct object of interest appearing in an image [37]. For instance, if two cells are present in an image, semantic segmentation would classify them as cells, and instance segmentation would identify each cell separately. Instance segmentation is more challenging than other pixel-level learning problems such as semantic segmentation, where each pixel can belong to a set of predefined groups (or classes), whereas in instance segmentation, the number of groups (instances) and their location are unknown a priori.

There are many segmentation techniques, which are now considered as *traditional* image processing (as opposed to the more recent *deep learning* approaches) and consist of algorithmic steps that allocate a pixel to a given class through a series of steps related to the characteristics of the pixel, say its intensity or relation to its neighbours. The simplest segmentation is that which assumes that pixels above a certain intensity level belong to a class, and those that do not belong to a different class. Selecting an intensity could be thought of as selecting an altitude that will distinguish *mountains* above that altitude against the lower level regions, i.e., the valleys. This is considered as *intensity level thresholding*, and it works well as long as the objects of interest are indeed of a different intensity as the background. This can be the assumed in fluorescence microscopy for instance.

A different approach to that of the thresholding would consider not the altitude, but the inclination of the region. To continue with topological analogies, consider a landscape (i.e., the intensity-based pixels), over which rain would fall. The water would follow the route downwards through which it could reach a lower region, i.e., a lake. Each point of the image would correspond to one route towards one and only one lake, and the image would be partitioned into catchment basins. This process is called *watershed* segmentation [38,39] and has been successfully used to segment tightly packed cells [40]. On the other hand, the detection of those regions with the steepest change is the basis of edge detection techniques [41,42], which are widely used in image processing.

Processing a large region on a pixel-per-pixel basis has several disadvantages: it is computationally expensive; it may suffer from noise; and there may be local bias, e.g., the intensity of a region may experience a smooth uniform variation, but still be a single region. A practical approach for these problems is the generation of larger, visually uniform regions or entities, which have been called *super-pixels* [43], based on different characteristics (contours, texture, brightness, etc.) of an image. Super-pixels are now widely used as a pre-processing, low-level tool that oversegments an image with the objective that one or more of the super-pixels will be a significant region of interest [44,45].

One other methodology that can be used for segmentation, as well as other tasks is the *active contour model* [46]. The active contour consists of a model, sometimes called a *snake* or *balloon*, which is initialised to a region of the image and deforms in a series of iterations to adapt and delineate the outline of objects of interest. There is an assumption that the object is known, but only approximately, and the contour will evolve, avoiding noise towards the object. Once detected, the contour can evolve and track an object that moves. Active contours are widely used and have been applied to problems of medical imaging such as brain segmentation [47] or histopathology [48].

The reader is referred to [49] for a classic review on image segmentation, [50] for a review on medical image segmentation and [51] for a review on cell segmentation.

The segmentation, identification and analysis of EM cellular images can be performed through manual processes [52,53,54], which can be distributed as *citizen science* where an *army of non-experts* [55,56] are recruited to provide non-expert human annotation, segmentation or classification through web-based interfaces (e.g., https://www.zooniverse.org/projects/h-spiers/etch-a-cell (Accessed on 28 May 2021)) [57]. Alternatively, computational approaches with traditional algorithms or deep learning approaches have been proposed to detect neuronal membrane and for mitosis detection in breast cancer [58], mitochondria [59,60], synapses [61] and proteins [62]. In particular, the segmentation of the plasma membrane of HeLa cells has been attempted either manually [63,64,65,66] or at a much lower resolution [67] (e.g., pixel resolutions around 312 nm, whilst in this work, 10 nm), which constitute a completely different problem. An alternative approach to a segmentation can be a plasma membrane *simulation* based on simulated *lipid particles* that distribute around the intensity of an image [68]. Whilst manual segmentation is very precise and exploits the power of the human visual system of an experienced researcher, this is extremely time consuming: manual delineation of one nuclear envelope can take around 30 h [69], and there may be significant intra- and inter-observer variability.

Besides the well-known limitations of deep learning architectures, of significant computational power, a large amount of training data and problems with unrelated datasets that show little value for unseen biological situations [70,71,72,73,74,75], the resolution of the EM datasets can enable or restrict their use for specific purposes. For instance, with a voxel resolution of 10 nm and a slice separation of 40 nm, it is possible to observe axons and dendrites well [76], yet when the voxel resolution is isotropic at 4 nm, an exquisite definition of macromolecular structures such as the Endoplasmic Reticulum (ER) and microtubules is visible [77]. A notable contribution is CEM500K [78], a very large dataset of images, pre-trained models and a curation pipeline for model building specific to EM. In addition, the specific nature of a semantic segmentation can allow traditional algorithms to provide satisfactory results, in some cases superior to deep learning approaches [79]. Previous work compared the algorithm used here to segment the nuclear envelope against active contours [69] and five deep learning architectures [79]. The active contour approach required manual seeding, which although not difficult to provide, still required manual intervention. However, the outcome (Jaccard similarity index = 75%) was not satisfactory and was probably due to sensitivity to local minima. In addition, it was significantly slower due to the iterative nature of the algorithm. The algorithm provided excellent results (AC = 99%, JI = 93%), which outperformed active contours and four deep learning architectures, specifically: VGG16 (93%, 90%), ResNet18 (94%, 88%), Inception-ResNet-v2 (94%, 89%) and U-Net (92%, 56%). The case of U-Net was interesting. The performance was significantly lower than the other approaches, and it was observed that U-Net was detecting and segmenting all the nuclei in the field-of-view, when the task was to segment just the central nucleus. It is well known that the training and tuning of deep learning architectures can alter the results significantly. Perhaps larger training datasets or the use of pre- and post-processing could improve the results of the deep learning approaches. However, the accuracy of the segmentation of the NE provided by the traditional segmentation algorithm was sufficiently good to employ as a step towards the segmentation of the plasma membrane.

This paper describes an extension to previous work that focused on the segmentation of the NE of a cell from a cropped volume [69,79]. In this work, individual HeLa cells and their nuclei were instance segmented in 3D. The cells were identified and selected from a volumetric stack of 518 slices with 8192 × 8192 pixels each. The number of cells to be identified and segmented can be selected as some cells will be close to the edges of the volume and will not appear complete. In order to segment an individual instance of one cell, regions of interest of 2000 × 2000 × 300 voxels, which contained a cell in the centre, were automatically cropped. The background and NE were automatically segmented, and the resulting regions became the input to a series of steps of morphological distance, watershed, morphological operations that segmented the cell of interest, i.e., the instance, from neighbouring cells. Thus, the contributions of this work were: (a) the automatic identification and cropping of volumetric regions of interest that contain individual cells and (b) the segmentation of the plasma membrane of a single cell and separating it from neighbouring cells. The rest of the manuscript is organised as follows: Section 2 describes the cell preparation, acquisition with SBF SEM and the algorithm to detect the cells, to crop individual cells into volumes and to segment the nuclei and plasma membranes. Section 3 presents the results and discussion. Different visualisations are presented in several figures to appreciate the details of the results. The limitations of the algorithm are explored. Finally, Section 4 presents conclusions and suggests future work.

All the code related to this work was performed in the programming environment of MATLAB^®^ (The MathWorks^TM^, Natick, MA, USA). The code, ground truth of one cell and EM images are available:

- https://github.com/reyesaldasoro/Hela-Cell-Segmentation.
- https://doi.org/10.5281/zenodo.4590903
- http://dx.doi.org/10.6019/EMPIAR-10094.

(Accessed on 28 May 2021).

## 2. Materials and Methods

### 2.1. Cell Preparation and Acquisition

HeLa cells were were prepared, embedded in Durcupan and observed with SBF SEM following the method of the National Centre for Microscopy and Imaging Research (NCMIR) [80]. SBF SEM data were collected using a 3View2XP (Gatan, Pleasanton, CA, USA) attached to a Sigma VP SEM (Zeiss, Cambridge, UK). In total, five-hundred eighteen 518 images of 8192 × 8192 pixels were acquired. The voxel size was 10×10×50 nm with intensity [0–255]. Figure 1a shows six EM images positioned within the 3D stack. Initially, the data were acquired at a higher bit-depth (32 bit or 16 bit), and after contrast/histogram adjustment, this was reduced to 8 bit [69]. Images are openly accessible via the EMPIAR [81] public image database (http://dx.doi.org/10.6019/EMPIAR-10094 Accessed on 28 May 2021).

### 2.2. Segmentation of Background and Identification of Cells

**Figure 2:**
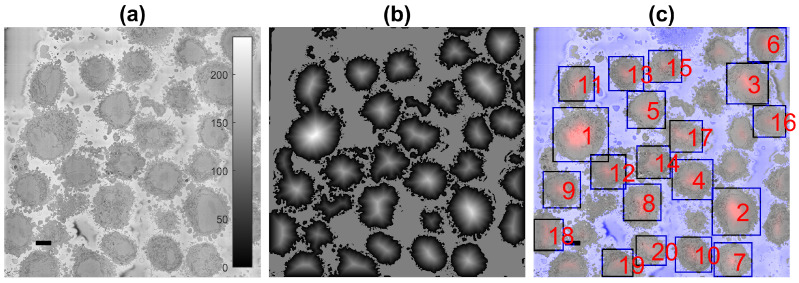
Automatic identification of cells from 8192 × 8192 images. (**a**) One representative slice with many HeLa cells. The scale bar corresponds to 5 μm. (**b**) Illustration of the detected background (grey constant shade) and distance transform (grey to white) that corresponds to the cells; the larger the cell is, the brighter the intensity of the transform. (**c**) Composite image of the slice as in (**a**), background as a purple shade and 20 detected cells, ranked in order of size. It should be noted that smaller cells were not selected as there was a limit of 20 in the present example.

One characteristic feature of the images is that the background, that is the resin in which cells have been embedded, tends to be brighter than the cells, and is fairly uniform (Figure 2a). This allows the segmentation of the background on the basis of intensity. Cells are identified by a combination of intensity thresholding using Otsu’s algorithm [82], the generation of super-pixels by detecting edges with a Canny edge detector [41] and morphological operators to clean the output (Figure 2b). It should be noted that an important limitation of the algorithm to identify cells is the presence of a brighter background. The background helps to identify individual cells and the proper delineation of the membrane, with its blebs and protrusions. When the presence of the background is limited, as with cells that are close to each other, the segmentation will separate cells from each other, but the protrusions, which may belong to either cell, may not be assigned as part of the cells. The presence of background is not a requirement for the nuclear envelope, as will be demonstrated below.

The segmentation of the background leads to the identification of the cells through a calculation of a distance transform. Moreover, the distance allows identifying the size of the cells as a larger cell will have pixels that are further away from the background (Figure 2b). This could be understood as a topological analogy where the distance transform produces an *altitude* map and each cell corresponds to a *hill*. The ranking of the cells follows the altitude in descending order (Figure 2c). In some cases, it is possible that a single cell will provide more than a single peak, i.e., a range in the topological analogy. The process to identify these peaks as belonging to a single cell is to proceed iteratively from the highest peak and discard any other peak within a certain distance around it.

The number of cells to be identified can be pre-defined, e.g., 20 cells for the example of Figure 2c. The identification is repeated for a number of slices of the 3D stack, and the centroids of the cells are located in 3D. Then, these centroids are linked vertically to identify which of them correspond to the same cell, as it should be noted that Cell 1 will always be the largest cell of the particular slice, and as the slices move up or down from what would be the equator of a cell, their size will change.

**Figure 3:**
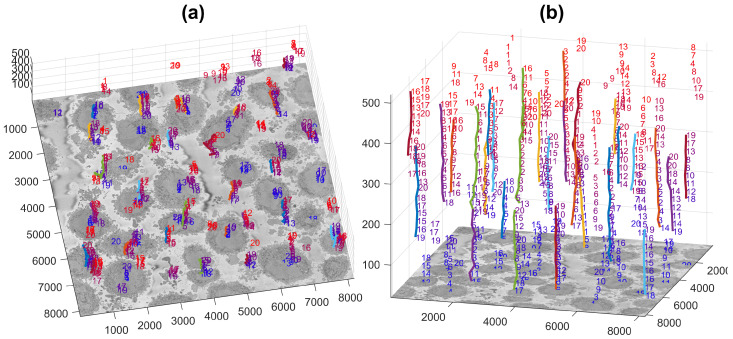
Centroids of cells that were identified per slice are displayed in three dimensions. Each number corresponds to the centroid of one cell that has been identified in a given slice. The numbers decrease according to the rank of the cell in that slice with 1 being the largest cell detected in that slice. The colour of the font varies from blue (lower slices) to red (higher slices) for visualisation purposes. A coloured line with a random colour is placed next to the centroids that were associated as a single cell. (**a**,**b**) show the same information from different points of view. The units of the axes are in voxels.

Figure 3 illustrates the centroids located at every 20 slices (i.e., 26 slices were analysed) with the number from their corresponding slice and a coloured line indicating the centroids that were linked as a single cell and posteriorly cropped into the regions of interest of (2000 × 2000 × 300) voxels, in which one cell was centred. It should be noted that the only the largest 20 cells were identified, with a few more smaller cells still present. These cells may be small in the current slice as they were close to the edges, i.e., the poles, but could be larger in other slices.

### 2.3. Semantic Segmentation of the Nuclear Envelope

The process of segmentation continues now for each cell cropped within the 2000 × 2000 × 300 region of interest identified in the previous steps. The methodology for the automated segmentation algorithm of the Nuclear Envelope (NE) has been published before [69], but for completeness is summarised in this section.

In order to remove high-frequency noise and therefore to improve segmentation, initially, all 518 HeLa cell EM images were low-pass filtered with a Gaussian kernel with size h=7 and standard deviation σ=2. Then, Canny edge detection [41] was used to determine the abrupt discontinuity in brightness or intensity changes between the NE and the neighbouring cytoplasm (outside the nucleus) and nucleoplasm (inside the nucleus).

The Canny edge detection resulted in some disjoint segments due to the NE variations in intensity, and these segments were connected by dilation using a distance map from the edges. All pixels within an adaptive distance, i.e., 5 pixels, which grew depending on the standard deviation of the Canny edge detector, were connected as a single edge. Those pixels that were not considered as edges were labelled as a series of *super-pixels*. The nucleus tended to be the larger and central super-pixel, but several morphological operators were required to remove regions in contact with the borders of the image, remove small regions, fill holes inside larger regions and close the jagged edges before identifying the nucleus and its surrounding NE.

To process a volume, the algorithm started from the central slice of the EM stack, e.g., the equator of the nucleus, and the image-processing algorithm exploited the volumetric nature of the data by propagating the segmentation of the NE of one slice to the next, up and down from the centre. The NE of a previous slice was used to check the connectivity of disjoint regions or *islands* separate from the main nuclear region. The algorithm proceeded in both directions and propagated the region labelled as nucleus to decide if a disjoint nuclear region in the neighbouring slices was connected above or below the current slice of analysis. When a segmented nuclear region overlapped with the previous nuclear segmentations, it was maintained; when there was no overlap, it was discarded.

### 2.4. Semantic Segmentation of the Plasma Membrane of a Cell

**Figure 4:**
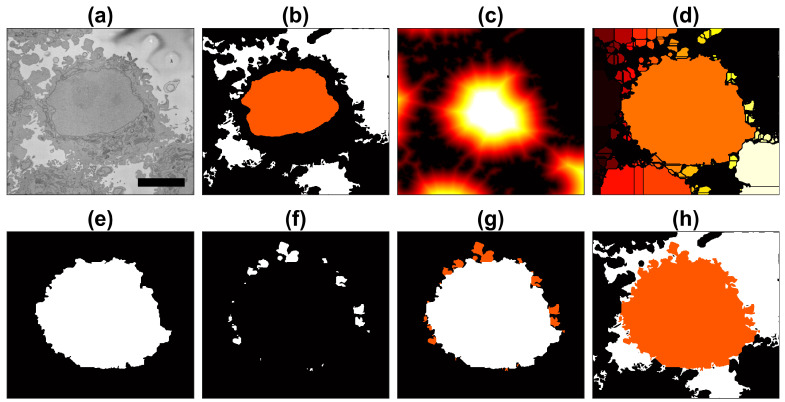
Illustration of the steps of the segmentation algorithm for a cell from neighbouring cells. (**a**) Region Of Interest (ROI) that contains one HeLa cell surrounded by background and other cells. (**b**) The algorithm starts with the nuclear region and background. (**c**) Distance transform from the background. (**d**) Watershed transformation on the distance transform; all regions in the background were removed. (**e**) Central region from the watershed. (**f**) Small regions that were contiguous to the central region. (**g**) Addition of small regions, i.e., membrane protuberances. (**h**) Final result of the cell with the background in white and neighbouring cells in black. A 5 μm scale bar is shown in (**a**).

The instance segmentation of one cell from its neighbours is a relatively simple process when the background and nuclei have been previously identified (Figure 4a,b). Since the current cell was cropped into a region of interest where the cell nucleus was positioned near the centre of a 2000 × 2000 × 300 voxel volume, the segmentation became an instance segmentation as the cell would be identified as a cell and different from other cells that surround it.

The distance transformation from the background (Figure 4c) grew around regions with cells, and since there would be one larger *hill* at the centre, the distance transformation can be segmented with a watershed algorithm [38]. The watershed is useful to separate one cell from other cells within the field-of-view. Watersheds are well-known for oversegmenting and other artefacts, so the central and largest region is selected as the cell (Figure 4d,e). This region was morphologically *opened* with large structural elements to remove protruding artefactual regions produced by the watershed (Figure 4e). This returned a fairly round cell, which would not include the natural protrusions (i.e., pseudopods or cell membrane extensions) of the cell. Thus, regions that were contiguous to this central region and surrounded by background were identified and merged with the cell (Figure 4f,g). The final segmentation of the cell with the inclusion of these natural protrusions or protuberances of the cell membrane can be observed in Figure 4h.

### 2.5. Quantitative Comparison

In order to assess the accuracy of the segmentation algorithm, two different pixel-based metrics were used: Accuracy (AC) and the Jaccard similarity Index (JI) [83]. Both metrics arise from the allocation of classes (nucleus, cell, background) to every pixel of an image and the correct or incorrect prediction of the class by the segmentation algorithm. For each pixel, four cases exist: True Positive (TP), which corresponds to pixels that were correctly predicted as a certain class (e.g., nucleus), True Negative (TN), False Positive (FP) and False Negative (FN). Thus, accuracy is calculated as:

(1) $$\mathrm{AC}=\frac{TP+TN}{TP+FN+TN+FP}$$

and the Jaccard similarity index is calculated as:

(2) $$\mathrm{JI}=\frac{TP}{TP+FP+FN}$$

It should be noted that JI is a more rigorous metric since the TNs, or correctly segmented background pixels, are not taken into account. A very small region of interest surrounded by a very large background could produce a very accurate result due to the presence of many TNs.

**Figure 5:**
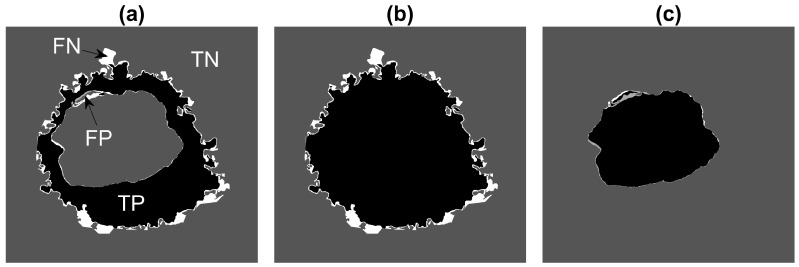
Illustration of the pixel-based metrics. True Positives (TPs, black), True Negatives (TNs, dark grey), false Positives (FPs, light grey) and False Negatives (FNs, white) are presented with increasing grey level intensity. (**a**) Cellular region excluding nucleus. (**b**) Entire cellular region. (**c**) Nucleus. FNs are far more common than FPs in (**a**,**b**) as some convoluted regions of the cell were not segmented.

The AC and JI metrics were calculated for three different scenarios: (a) the segmentation of the cell excluding the nucleus, (b) the cell including the nucleus and (c) the nucleus only. Figure 5 illustrates these cases for a sample slice of the data.

## 3. Results and Discussion

A volume of interest of 8192 × 8192 × 518 voxels was analysed as previously described. The cell identification process was run for 26 slices, i.e., every 20 slices. At each slice, the algorithm was set to detect 20 cells. When the results of all slices were linked, these identified 30 cells in total. The only manual intervention of this process was to select the number of cells per slice and the number of slices to be analysed. Thirty cells automatically identified were comparable to those analysed through citizen science approaches, i.e., 18 [57].

**Figure 6:**
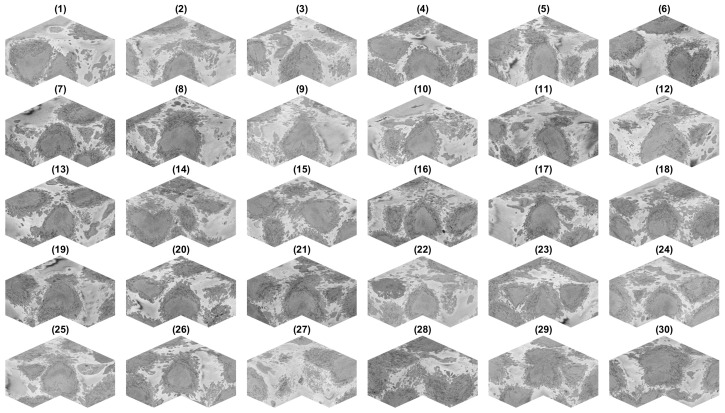
ROIs cropped from the volume. Thirty regions of interest were detected and cropped. For each region of interest, a corner was removed to show the cell, which should be centred. It should be noted that some cells were not in the centre, but rather positioned towards the bottom of the volume (e.g., 1), the top (e.g., 30) or the sides (e.g., 6,14,15,27,28).

These cells were automatically cropped into individual ROIs and saved in separate folders as 300 2000 × 2000 Tagged Image File Format (TIFF) images. The ROIs are illustrated in Figure 6.

It was observed that some of the ROIs were close to the top or bottom (e.g., ROIs 1, 2, 29, 30) and thus would contain only part of a cell, and the rest would be outside the field-of-view in the vertical direction. Similarly, the centroids of other ROIs were close to the edges of the volume and thus were not centred, but rather positioned towards a side of the ROI (e.g., 6, 14). Since the actual centroid was recorded, it was possible to determine how far away (i.e., absolute distance) the centroids were from the edges of the initial 8192 × 8192 × 518 volume. The centroid of ROIs 6, 14, 15, 27 and 28 were less than 500 pixels from the edge. Considering that these ROIs were 2000 pixels wide, this indicated that the centroid was considerably distant from the centre, closer to the edges than to the centre of the 2000 × 2000 × 300 volume. It should be noted that one assumption of the algorithm for nuclei segmentation was that the nuclei were centred in the ROI. In addition, being located away from the centre allowed the possibility of two partial cells occurring in the same ROI. Thus, six ROIs (6, 14, 15, 27, 28) were discarded and not further processed. This decision was made manually, but it could be automated by determining a certain margin, e.g., 500 pixels or 25% of the width, from the edges. Alternatively, it could be possible that instead of fixing the size of the ROI to be 2000 × 2000 × 300, a different size could be allocated so that the centroid of the cell is always at the centre of the volume.

The situation of cells that were close to the top (29, 30) and bottom (1, 2) was slightly different. Whilst the cell was not centred *vertically*, it was still centred *horizontally* within the plane of the images, and it could be segmented successfully if the central slice, corresponding to the equator, to be processed was adjusted with the centroid location. The segmentation would not be of a complete cell, but only part of it, so the segmentation may appear as a *hat* when it is in the lower section of the volume or a *bowl* if it is in the upper section. If metrics such as volume are to be obtained, then these cases are not to be used. In this paper, we were interested in the observation, rather than a quantification, and thus, these cells were not discarded from further processing.

**Figure 7:**
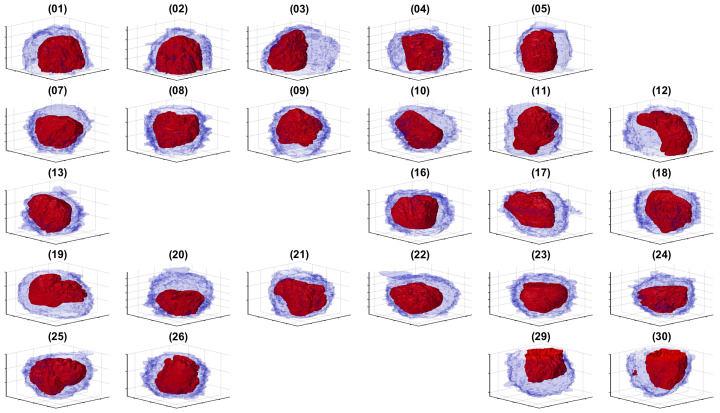
Rendering of the cell and nuclear envelope (NE) of 25 cells. For each case, the NE is rendered in red without transparency, and the cell membrane is rendered in blue with transparency. The cells in ROIs 6,14,15,27 and 28 were located on the edges of the volume, and the centroids were too close to the edges and thus discarded. For comparison purposes, the cells were placed in the same locations as in Figure 6, and the ROIs that were discarded are blank.

**Figure 8:**
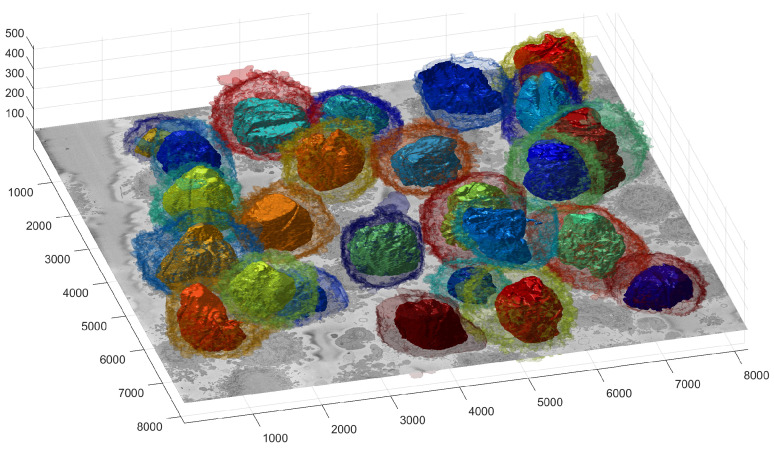
Illustration of the segmentation of 25 cells and Nuclear Envelopes (NEs). The cells were segmented from a 8192 × 8192 × 518 voxel region. Slice Number 100 out of 518 is displayed for context. All nuclei are shown solid, and all cell membranes are shown as transparent; colours have been assigned randomly for visualisation purposes. The units of the axes are in voxels.

The cells and nuclei in the remaining 25 ROIs were segmented and are displayed separately in Figure 7 and together in Figure 8 with transparent membranes and solid NEs. Colours were assigned randomly in Figure 8 for visualisation purposes together with one slice of 8192 × 8192 pixels to give a reference.

The positions of Figure 6 are maintained in Figure 7 to facilitate the comparison between the ROIs and the segmentations. Whilst at this resolution, it was not easy to observe the details, it can be seen that the 25 cells and nuclei were successfully segmented. Geometric features from these surfaces could be extracted if different populations of cells, for instance treated/untreated, wild-type/mutant, healthy/infected were to be compared statistically. We did not conduct this analysis as all these cells corresponded to a single experimental condition.

**Figure 9:**
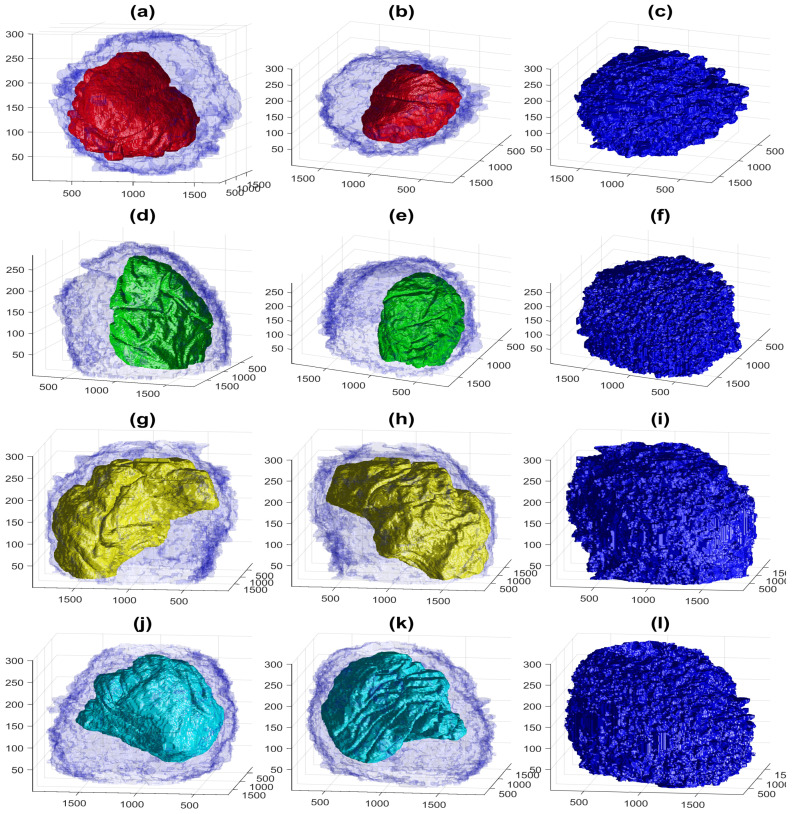
Four examples of the volumetric reconstruction of the NEs and the cell membranes of HeLa cells. In all cases, each row corresponds to a single cell observed from different view points. The left and centre columns show the cell membrane as transparent. The right column is the cell membrane without transparency from the same view point as the centre column. The volume of interest is 2000 × 2000 × 300 voxels, and the units of the axes are in voxels. (**a**–**c**) ROI 23; the NE is shown in red and the cell in blue. Notice the relative smoothness except for one groove along the cell and the concentration on the lower part of the cell. (**d**–**f**) ROI 3; the NE is shown in green. Notice the ruggedness of the NE with numerous grooves and the concentration of the nucleus towards one side of the cell. (**g**–**i**) ROI 12; NE is shown in yellow. Notice the distribution of the nucleus concentrated on the upper part of the cell. (**j**–**l**) ROI 19; NE is shown in cyan. The surface of the NEs appears more distinctive than those of the cells.

**Figure 10:**
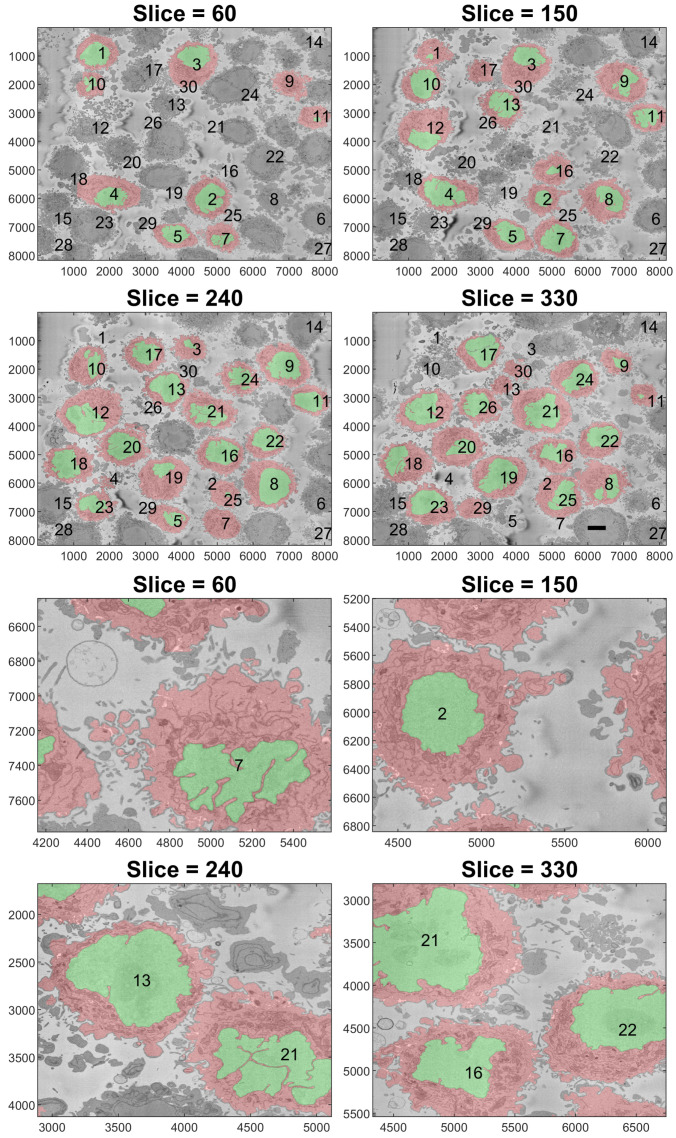
Final illustration of the results in four representative slices. Cells are highlighted with a red shade, and the nuclei are highlighted with a green shade. Numbers were added to aid the localisation of the particular cell. Notice that some of the numbers corresponded to cells that were not visible in that particular slice. The units of the axes are in pixels, and a scale bar indicating 5 μm is shown in Slice 330 on the top. The bottom shows a magnified version of the slices.

To better observe the details, the rendering of four cells is shown in Figure 9. The left and central columns show the cell membrane rendered with high transparency and the NE rendered as a solid surface from two different points of view. The right column shows the cell membrane without transparency from the same point of view as the central column. The first interesting observation was that the nuclei seemed to be far more different than the cells themselves in terms of the smoothness or ruggedness of the surface and the distribution of the nucleus within the cell. The first cell (a,b), rendered in red, was relatively smooth whilst the other cells have far more grooves on the surface. It is also interesting to observe that the nucleus did not appear to be in the same position for these cells, leaving space in the top (b), bottom (h,k) and roughly even (e). These characteristics, the uneven distribution of the nuclei and their variability as compared with the cells, are also visible in Figure 10, which shows the results overlaid on four slices of the data with nuclei with a green shade and cells with a red shade. Numbers to identify the ROIs were added, but it should be noted that some cells were not visible within certain slices and may be positioned over other cells not included in the analysis. Some cells (e.g., 3, 8, 12, 19) appeared to be polarised to a side with the perinuclear region with numerous organelles visible. The results shown in Figure 10 illustrated boundaries between several cells well, and the complexity of the plasma membrane can be appreciated.

One final observation of Figure 10 is that there was one cell that was not identified by the algorithm; the cell in the centre of Slice 240 surrounded by Cells 13, 21, 16, 19 and 21. This may be due to two factors. First, the algorithm was selected to identify only 20 cells per slice. Slice 240 showed 19 cells, and it should be remembered that Cells 6, 14, 15, 27 and 28 were discarded. Thus, it may be that by using a larger number per slice, e.g., 30, this cell would have been selected. Second, that cell in particular was rather *flat* in the vertical dimension if compared with Cells 13 and 16, which were visible in Slices 150, 240 and 330. Since the algorithm ranked cells by their size, the cell may have been smaller in size and thus not considered. Again, by extending the number of cells per slice, this could include this cell and others in the analysis.

**Figure 11:**
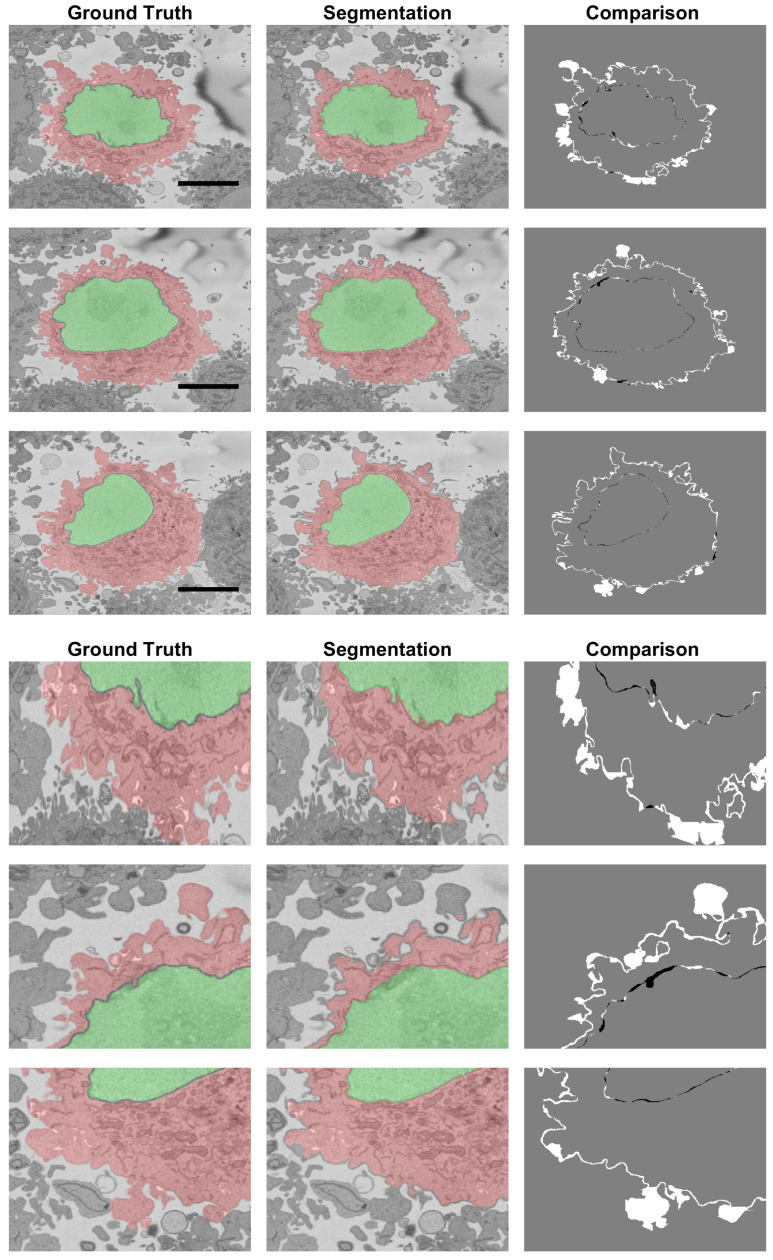
Comparison between the Ground Truth (GT) and the segmentation result obtained from the segmentation algorithm shown in three slices of the stack. The left column illustrates the GT with shades of green for the nucleus and shades of red for the cell. The centre column shows the result of the segmentation algorithm. The right column shows the comparison between the GT and the results with FNs in white, FPs in black and both TPs and TNs in grey. Large white regions correspond to the distinction between neighbouring cells. A 5 μm scale bar is shown in the GTs on the top, and a magnification is shown below.

The results for the cell for which the ground truth for the NE and cell membrane was available were the following: for the cell excluding the nucleus AC=0.9629, JI=0.8094, for the cell and the nucleus AC=0.9655, JI=0.8711 and for the nucleus alone AC=0.9975, JI=0.9665. The algorithm to segment the nucleus provided excellent results, and it had previously been reported that it outperformed several deep learning architectures [79]. The small differences between the segmented nucleus and that of a manual expert segmentation were due mainly to the calculations of the thickness of the NE and small invaginations (Figure 11, right column). As would be expected due to the complexity of the cell membrane, the values for the cell were relatively lower than those of the NE. Figure 11 shows that there were relatively few FPs in comparison to the FNs. This was a characteristic of the segmentation algorithm, i.e., it was *cautious* to include the protuberances that surrounded the cell. These regions are better observed in the bottom section of Figure 11, which zooms in and illustrates regions where these FNs appear. It can be perceived that in some cases, it was difficult to decide if a small region belonged to the cell of interest or to the neighbouring cells. It is well known that it is difficult to establish the *truth*, and there could be significant inter- and intra-observer variability [57].

**Figure 12:**
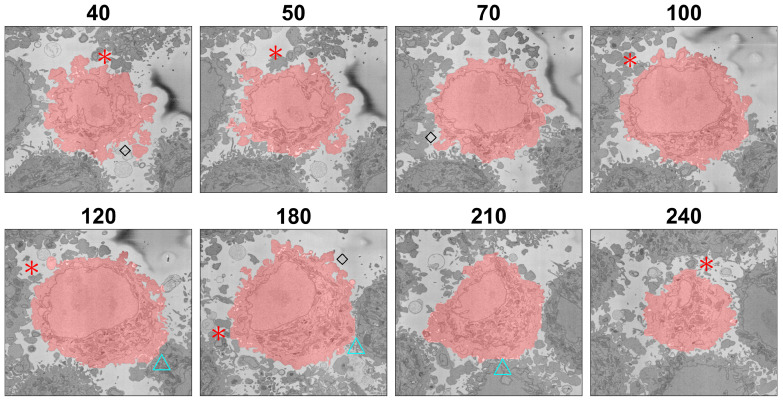
Illustration of the segmentation at several slices of one cell. The segmentation is indicated with a red shade over the cell. Red asterisks indicate regions where the segmentation did not include protuberances that could belong either to the cell or to neighbouring cells. Blue triangles indicate regions where two cells were close together and the segmentation tended to a straight line between the cells. Black diamonds indicate convoluted protuberances that were correctly segmented. The slice number relative to the stack of 300 is indicated above each slice.

This situation is further illustrated in Figure 12, which shows several slices of the volumetric stack. Asterisks were placed next to regions that could belong to either the cell of interest or neighbouring cells. In all the cases illustrated, the algorithm did not include these as part of the cell. The risk of modifying the algorithm to include the protuberances that were not included would be that the cell could grow into neighbouring cells. Protuberances surrounded mostly by background and not so close to other cells were better segmented as indicated by black diamonds. If the cell surfaces were to be analysed for counting filopodia or to distinguish filopodia from lamellipodia, the algorithm would have to be refined.

The segmentation of one cell from other neighbouring cells required the presence of a certain amount of background surrounding the cell, as this was used to form the distance map, which would later be segmented with the watershed. When two cells were very close together and there was no background, or very little of it, in that region, the segmentation tended to be a straight line that bisected the cell with its neighbouring cells. This is illustrated in Figure 12 with cyan triangles next to those boundaries. As mentioned earlier, false negatives, i.e., the convoluted regions not included, outnumbered false positives; false negatives constituted 3.46% of voxels, whilst false positives were only 0.25% for the cell without the nucleus and 3.31% and 0.14% for the cell including the nucleus. For reference, the true positives were 15.7/23.33% and the true negatives 80.5/73.2%. Thus, the algorithm was a lower-bound estimation of the cell and was not including as part of the cell regions that could belong to either of two neighbouring cells, around 3% for the one cell for which the GT was available. Again, if a very precise cell–cell interaction based on the surfaces were of interest, the algorithm would have to be refined. Still for many other applications such as the measurement of volume, position and polarisation, this algorithm provided a very good approximation of the cell membrane and an excellent segmentation of the NE. Another application where this algorithm can be used is for counting mitochondria or other structures on a *per-cell basis*, so that each instance of a mitochondrion can be allocated to the correct cell.

As an indication of the computational complexity, the times to run the following processes were recorded in an Alienware m15 R3 Laptop with an Intel^®^ Core™i9-10980HK CPU, 2.40 GHz with 32 GB RAM and running MATLAB ^®^ (MathWorks™, Natick, MA, USA) Version: 9.8.0.1417392 (R2020a): (1) Detection of 20 cells in 26 slices and joining to identify 30 cells, 48 s, (2) cropping 30 ROIs and saving 300 TIFF images in 30 folders 9.1 min (18.2 s/ROI) and (3) segmentation of one nuclei 14.9 min (2.98 s/slice, (4) segmentation of one cell 13.2 min (2.64 s/slice). These times were significantly faster than manual segmentation, which can take around 30 h to segment one NE alone [69].

**Figure 13:**
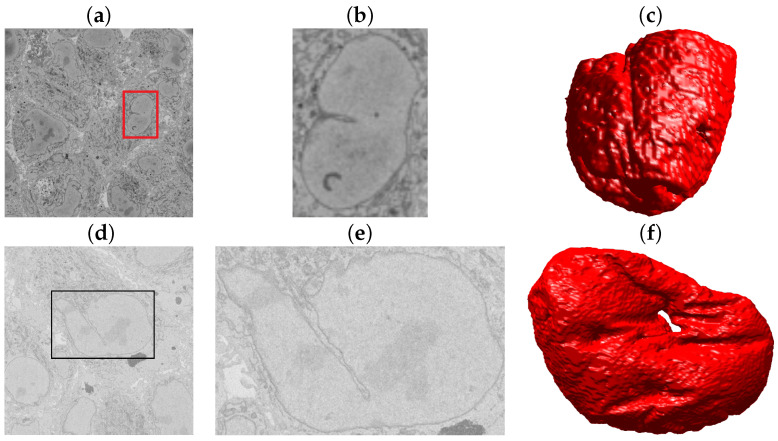
Illustration of the Serial Block Face Scanning Electron Microscope (SBF SEM) images containing monolayers of Chlamydia trachomatis-infected HeLa cells. (**a**) A representative image from the Cell Image Library CIL50051 dataset. The volume has 3200×3200×413 voxels, and the voxel size is 3.6×3.6×60 nm. (**b**) An ROI with one nucleus, which corresponds to the red box in (**a**). (**c**) Rendering of the NE of this cell. (**d**) One representative image from the Cell Image Library CIL50061 dataset. The set has 2435×2489×406 voxels and a voxel size 8.6×8.6×60 nm. (**e**) An ROI with one nucleus corresponding to the black box in (**d**). (**f**) Rendering of the NE of this cell.

So far, one of the strongest limitations of the algorithm was the presence of a bright background. To further test the segmentation algorithms, we identified cells with different settings from the 8192 × 8192 × 518 volume previously described. Two publicly available EM datasets from the Cell Image Library (CIL) (http://cellimagelibrary.org/images/50051 and http://cellimagelibrary.org/images/50061 Accessed on 28 May 2021) were analysed. These sets consisted of Chlamydia trachomatis-infected HeLa cells also embedded in Durcupan and acquired with SBF SEM using a Gatan automated 3View system (Gatan Inc.) [84]. The intensities were remarkably different from the previous dataset, as illustrated in Figure 13a,d. Notice especially the low contrast of (d). It was observed that the closeness between cells resulted in a very limited background, and thus, the analysis was restricted to the NE from manually cropped ROIs (Figure 13b,e). The nuclei were segmented, and the results were considered very good through a visual inspection (Figure 13c,f).

**Figure 14:**
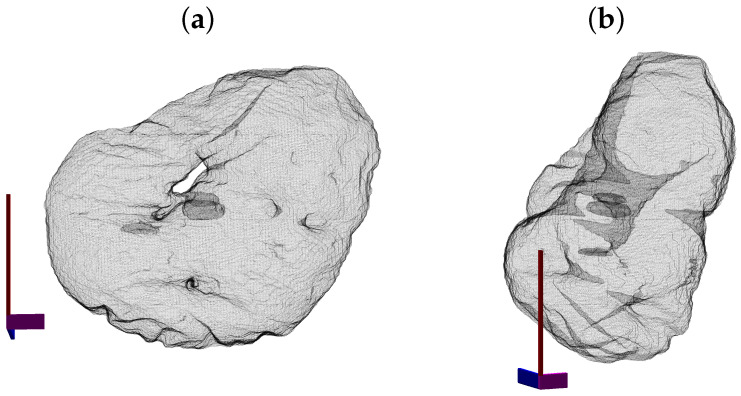
Illustration of the nuclear envelope from the dataset CIL50051. The surface is displayed as a mesh with transparency to show the hole of the nuclear envelope (**a**) and the crevices that go deep inside the nucleus. Notice in (**b**) how these invaginations nearly connect separate sides of the NE.

One further observation was relevant. The surface in Figure 13f showed a hole so it was interesting to display the surface of the NE in a different way with the NE rendered as a mesh with no face colour, edges in black and with transparency (Figure 14). Axis were added for reference. Besides the evident hole, the NE had other deep crevices that nearly connected two opposite sides of the NE, as can be observed in the lower part of (b).

## 4. Conclusions

In this paper, an algorithm to segment instances of HeLa cells as observed with electron microscopy was described. The algorithm followed a sequence of image processing steps that identified a background, located cells in three dimensions, cropped selected cells into separate regions of interest, segmented the nuclei and, finally, segmented the plasma membrane. The algorithm can be run automatically, and only some parameters (number of cell per slice, number of slices, cells to be discarded) were set manually. The results of the algorithm were assessed computationally for one cell for which the ground truth was available and visually for the other 24 cells segmented. The results of the algorithm were very accurate for the segmentation of the nuclear envelope, as had been demonstrated previously against active contours [69] and several deep learning architectures [79]. The segmentation results of the plasma membrane were lower, as would be expected due to the significantly more complex geometry. Even for experienced researchers, the plasma membrane is far more complicated than the nuclear envelope.

Some of the advantages of the algorithm described are the following: (a) The algorithm does not need a large number of training samples. (b) The algorithm is fast as compared with the time that would be required to train any deep learning approach. (c) The algorithm can be customised in the case that only a few cells are of interest. (d) The algorithm could provide the starting point for further research, for instance: if sub-cellular structures such as mitochondria are of interest, this algorithm could restrict the region to search for them by excluding the regions outside the cell and inside the nuclei. (e) The algorithm could be used simply to identify regions of interest of a single cell and crop them automatically.

The algorithm has some limitations that have been discussed above. The most important are the following: (a) the presence of a bright background that allows the distinction of the cells (whilst it was shown that this was not a limitation to segment the nuclear envelope, it would be difficult to segment the plasma membrane without the bright background of the Durcupan resin); (b) the *cautious* delineation of the membrane, where some regions that could belong to either a cell or its neighbour were not included in either. For the cell of interest, these regions represented around 3% of the cell. Assuming that these underrepresentations would be consistent along all cells, comparisons between cells such as volume, surfaces or ratios could still reveal differences among populations. Thus, for for a general analysis of the cell surface, the algorithm provided good results, and if the details of the protrusions of the cell were required, further work could consider a post-analysis of all regions not included once all cells were segmented.

Future work could focus on several lines. (1) A geometric analysis of the cell and nuclear surfaces could provide interesting insights into why some cells appeared smooth and others ruffled. (2) The segmentation of other organelles, for instance mitochondria and Golgi apparatus, could follow once the nucleus and extracellular regions are identified. (3) If further data were to be collected, many experiments could be performed and the cells compared. For example, cells could be exposed to potential therapeutic interventions and compared against untreated cells. (4) The segmentation could be performed not only in the slice dimension (i.e., x,y), but also in orthogonal projections (x,z and y,z) or a tri-axial approach, as suggested in [57]. This may improve the difficult sections at the top and bottom of the cells.

## Data Availability

The code associated with this article is available as open source at https://github.com/reyesaldasoro/Hela-Cell-Segmentation, and the data are available at http://dx.doi.org/10.6019/EMPIAR-10094, and https://doi.org/10.5281/zenodo.4590903 (Accessed on 28 May 2021).

## Abbreviations and Abbreviations

### Abbreviations

EM	Electron Microscopy
ER	Endoplasmic Reticulum
TP	True Positive
TN	True Negative
FP	False Positive
FN	False Negative
GT	Ground Truth
AC	Accuracy
JI	Jaccard Index
NCMIR	National Centre for Microscopy and Imaging Research
CIL	Cell Image Library
NE	Nuclear Envelope
ROI	Region Of Interest
SBF SEM	Scanning Block Face Electron Microscopy
TIFF	Tagged Image File Format
